# Topographic models for predicting malaria vector breeding habitats: potential tools for vector control managers

**DOI:** 10.1186/1756-3305-6-14

**Published:** 2013-01-16

**Authors:** Jephtha C Nmor, Toshihiko Sunahara, Kensuke Goto, Kyoko Futami, George Sonye, Peter Akweywa, Gabriel Dida, Noboru Minakawa

**Affiliations:** 1 Department of Vector Ecology and Environment, Institute of Tropical Medicine (NEKKEN), Nagasaki University, Nagasaki, Japan; 2 Department of Animal and Environmental Biology, Delta State University, Abraka, Nigeria; 3 Department of Eco-epidemiology, Institute of Tropical Medicine (NEKKEN), Nagasaki University, Nagasaki, Japan; 4 Ability to Solve by Knowledge, Community Project, Mbita, Kenya; 5 NUITM-KEMRI Research Program, Kenya Medical Research Institute, Nairobi, Kenya; 6 School of Public Health, Maseno University, Maseno, Kenya; 7 Global Centre of Excellence Program, Institute of Tropical Medicine (NEKKEN), Nagasaki University, Nagasaki, Japan

**Keywords:** Malaria, *Anopheles*, Breeding habitats, Prediction, Topographic models, Kenya, Africa

## Abstract

**Background:**

Identification of malaria vector breeding sites can enhance control activities. Although associations between malaria vector breeding sites and topography are well recognized, practical models that predict breeding sites from topographic information are lacking. We used topographic variables derived from remotely sensed Digital Elevation Models (DEMs) to model the breeding sites of malaria vectors. We further compared the predictive strength of two different DEMs and evaluated the predictability of various habitat types inhabited by *Anopheles* larvae.

**Methods:**

Using GIS techniques, topographic variables were extracted from two DEMs: 1) Shuttle Radar Topography Mission 3 (SRTM3, 90-m resolution) and 2) the Advanced Spaceborne Thermal Emission Reflection Radiometer Global DEM (ASTER, 30-m resolution). We used data on breeding sites from an extensive field survey conducted on an island in western Kenya in 2006. Topographic variables were extracted for 826 breeding sites and for 4520 negative points that were randomly assigned. Logistic regression modelling was applied to characterize topographic features of the malaria vector breeding sites and predict their locations. Model accuracy was evaluated using the area under the receiver operating characteristics curve (AUC).

**Results:**

All topographic variables derived from both DEMs were significantly correlated with breeding habitats except for the aspect of SRTM. The magnitude and direction of correlation for each variable were similar in the two DEMs. Multivariate models for SRTM and ASTER showed similar levels of fit indicated by Akaike information criterion (3959.3 and 3972.7, respectively), though the former was slightly better than the latter. The accuracy of prediction indicated by AUC was also similar in SRTM (0.758) and ASTER (0.755) in the training site. In the testing site, both SRTM and ASTER models showed higher AUC in the testing sites than in the training site (0.829 and 0.799, respectively). The predictability of habitat types varied. Drains, foot-prints, puddles and swamp habitat types were most predictable.

**Conclusions:**

Both SRTM and ASTER models had similar predictive potentials, which were sufficiently accurate to predict vector habitats. The free availability of these DEMs suggests that topographic predictive models could be widely used by vector control managers in Africa to complement malaria control strategies.

## Background

Human malaria is the most serious parasitic disease in the tropics. The present global malaria control strategy using long-lasting insecticidal nets (LLINs), indoor-residual spraying (IRS), and artemisinin-combination therapies (ACTs) have decreased morbidity and mortality due to malaria worldwide [1,2]. For further reduction in malaria transmission, supplemental strategies to LLINs, IRS, and ACTs are essential [2-4].

Targeting the immature stage may be a possible supplemental vector-control strategy, because LLINs and IRS are used to kill or repel only adult mosquitoes [5-11]. In recent larval control trials in Africa, successful results with hand-applied insecticides were limited to settings where mosquito larval habitats were well defined and not extensive [2], suggesting that defining target areas for larval control is essential. However, it is generally not an easy task to identify larval habitats over a large area. Extensive surveys of breeding sites are expensive, time-consuming, and labor-intensive, thus not feasible in countries with limited resources. Therefore, it would be beneficial to have practical models that can predict the locations of malaria vector breeding sites from easily obtainable information.

A few attempts have been made to predict malaria vector breeding sites based on remote sensing and topographic information [12,13]. Mushinzimana *et al.*[12] modelled the presence of larval habitats in Kenyan highlands using land-cover variables derived from remote sensing (LANDSAT, IKONOS, and aerial photo) images and topographic variables. Their model predicted larval habitats with nearly 80% sensitivity. Clennon *et al.*[13] examined various combinations of land-cover and topographic variables to predict larval habitats in southern Zambia. Their model, using land-cover variables derived from LANDSAT imagery and topographic variables derived from digital elevation models (DEMs), successfully predicted the occurrence of aquatic sites and larval habitats of malaria vectors. Similar models have been developed to predict areas with risk of malaria infection using land-cover and topographic variables [14,15].

These modeling approaches are effective for areas for which such land-cover and topographic information are available. However, it may not be generally practical to develop complex models using land-cover and topography variables, because satellite imagery and high-quality aerial photographs are sometimes not available, expensive, or not useful owing to cloud formations. Moreover, land-cover classification is a tedious and time-consuming task. On the other hand, topographic information is now freely available as DEMs for nearly the entire world and can be processed with free software. Given that, topography has fundamental importance in controlling surface water flow and pooling, it should have potential for predicting areas where suitable water bodies for malaria vector breeding would form. In previous studies, topographic variables have frequently been identified as important predictors of high-risk areas for malaria infection [14,16,17] and vector breeding sites [12,13,17].

In this study, we developed practical models that require only topographic information to predict the location of malaria vector breeding sites with acceptable accuracy. We used the results of an extensive survey of an entire large island in Lake Victoria. The island consists of various areas with differing topographic features, such as mountain peaks, cliffs, gentle slopes, streams, plains, swamps, etc.; thus, we expect that our model will be widely applicable to various environments with a range of topographic features.

## Methods

### Study area

**Figure 1:**
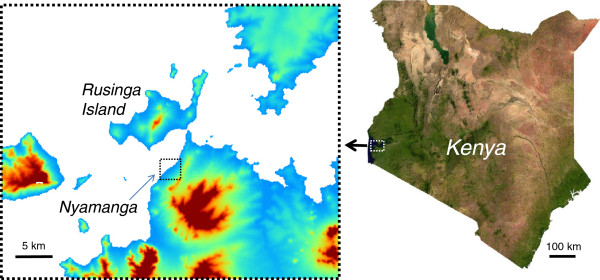
**Location of the study area.** (Right) Map of Kenya showing the location of the study area. (Left) Study area showing the training site (Rusinga) and the testing site (Nyamanga).

The primary study area covered the entire area (42 km^2^) of Rusinga Island, Mbita District in western Kenya (Figure 1). The island is the second-largest island in the Kenyan part of Lake Victoria and has been extensively deforested and cultivated. Streams are seasonal, and the main water source for the population is the lake. The rainfall pattern in the area is bimodal, with an extended rainy season occurring from March through May, and a shorter rainy season around November. In 1983, the island was connected to the mainland with a 200-m-long causeway [18]. To test the accuracy of the models developed based on the island, we considered another study area called Nyamanga located on the adjacent main land (Figure 1). The extent of this area was approximately 9 km^2^. Within both sites, most houses are constructed of a stick framework plastered with a mixture of mud and cow dung, with a corrugated iron roof. Few houses have more than two rooms. The majority of residents belong to the Luo ethnic group. Although Dholuo is the main language spoken, many residents speak English and Kiswahili. The main economic activities are fishing and farming.

### Mosquito larval habitat survey

An extensive survey of mosquito larval habitats covering the entire area of Rusinga Island was conducted in April 2006. Prior to the field survey, the habitat information on Rusinga Island was provided by the community based malaria control project [18]. Field assistants visited the breeding sites that had been monitored by the project members and examined for anopheline larvae. The assistants also searched for other water pools throughout the island. Each potential breeding site was examined for anopheline larvae using a standard mosquito dipper (350 ml; BioQuip Products, Rancho Dominguez, California, USA). Field assistants dipped a maximum of 50 times within each site. Breeding sites were categorized into tire tracks, footprints, drains and ditches, swamps, riverbeds, and puddles, artificial containers and holes, and tree holes. When multiple footprints were present in an area, they were considered as a single site. Habitats within the lake were excluded from the survey [19-22]. Artificial holes and containers, and tree holes were excluded in the model development because it is unlikely that formation of these habitats is affected by topographic variables. The coordinates of each site with anopheline larvae were recorded using a global positioning system (GPS). Anopheline larvae were not identified to species in this survey. However, previous studies have found that anopheline species from non-lake habitats in this area are mainly members of the *Anopheles gambiae* complex and the *Anopheles funestus* complex, which are important malaria vectors [19-23]. Supplementary data were collected in the course of a longitudinal survey in Nyamanga in 2010. Before the longitudinal survey, we identified 160 potential breeding sites at which we confirmed anopheline larvae or we considered that larvae likely occurred. These sites in Nyamanga had been surveyed routinely by field assistants. The sites where the presence of anopheline larvae was confirmed in May 2010 were used as positive sites in validation of the models.

### Digital elevation models (DEMs)

We used two digital elevation models (DEMs): 1) the Shuttle Radar Topography Mission 3 (SRTM3, 90-m resolution) DEM and 2) the Advanced Spaceborne Thermal Emission Reflection Radiometer Global DEM (ASTER GDEM, 30-m resolution). The SRTM DEM was collected during a space shuttle mission in 2000 using a multi-frequency and multi-polarization radar system. The absolute vertical and horizontal accuracies were set to ≤ 10 m and ≤ 20 m at the 90% level, respectively [24]. The data covers land surface between 60 degree N and 54 degree S and can be obtained freely from the National Aeronautics and Space Administration (NASA) web-site [24]. ASTER GDEM was generated from a large amount of ASTER images by automated processing using a stereo correlation method [25]. The vertical and horizontal accuracies estimated for the ASTER GDEM prior to its production were 20 meters and 30 meters respectively (both at 95% confidence level) [25]. This DEM covers land surface between 83 degree N to 83 degree S, and can also be downloaded freely [25]. More detailed technical information on these DEMs is available in the following websites [24,25].

Both DEMs are available in geographic coordinates (latitude and longitude in decimal degrees). However, for easier interpretation of the spatial scale in metric units, we converted both DEMs to the Universal Transverse Mercator (UTM) projection system (Zone 36 South) using the Systems for Automated-Geoscientific Analyses (SAGA) [26].

### Pseudo-absence

Although a predictive model is often, and perhaps best, built using techniques such as logistic regression modeling that relies on both presence and absence data [27,28], in our study, absence data were unavailable because the survey was designed to report only positive breeding sites. As an alternative, we generated random points within the study area and treated them as negative cases. Theoretically, the statistical power would be greater with larger numbers of pseudo-negatives [29]. However, as this study uses grid data with finite resolutions, multiple points in the same grid would be redundant. There are approximately 5,000 grids on Rusinga Island in SRTM DEM with 90 m resolution and the number of natural larval habitats was 826 (See Results). Therefore, we attempted to generate an approximately 5 times larger number of pseudo-absence points compared with the positive points, so that the total number of the points is close to 5,000. Using Microsoft Excel 2007, we initially generated 20,000 coordinates located within the extent of the island. The elevation of each point was then extracted from the SRTM DEM using SAGA [26]. Points with elevation below 1135 m were regarded as being on the lake and were therefore removed. Then points on another small island and the cape of the main-land were manually removed. The random points which are very close to the observed positive points are likely to have the same or very similar topographic features as the nearest positive sites, which may lessen the correlation between topographic variable and the likely occurrence of the habitats. Therefore, we removed the random points within 50 m from the positive sites. The cut-off distance of 50 m was chosen following Mushizimana *et al.*[12]. By this procedure, approximately 10% of the random points on the islands were removed. Thus, we obtained 4524 random points on the island that were at least 50 m distant from positive sites and treated them as negative points. The use of such pseudo-absence data in modeling is a recognized technique [30-32].

### Topographic variables

Eight topographic variables determined by local structure that potentially influence water content were extracted from the DEMs using the Terrain Analysis module of SAGA. First, the DEMs were smoothed to fill in isolated elevation pits (or spikes), which typically represent errors or areas of internal drainage that interrupt the estimate of water flow (Pre-processing, Fill Sink; Planchon and Darboux, 2001) [33]. Basic Terrain Analysis (BTA) was applied to the pre-processed DEM. Of the 11 variables derived from BTA, 6 were used in the analysis: slope, aspect, plan curvature, profile curvature, convergence index, and wetness index. In addition, two different scales of topographic position index (TPI) were calculated. In total, 9 variables including pre-processed elevation were derived from each DEM and examined as model predictors. The implications of the variables in terms of surface water accumulation are summarized below.

Elevation is a fundamental physical parameter defining soil-water gravitational potential energy [34] and is the primary influence on water movement throughout a landscape as well as within drainage channels. To enhance the applicability of our models to other areas with different elevation ranges, we converted the original elevation to relative elevations defined as the original elevation minus the lowest elevation in the area. The elevation of the surface of Lake Victoria was set as the lowest elevation for this analysis.

Slope is a measure of the change in elevation over a certain distance, or the difference in elevation between neighbouring cells, expressed as an angle from 0 to 90º. Slope has a strong influence on overland and subsurface flow velocity, drainage, and accumulation of water [35,36].

The aspect of a land surface is the orientation that the slope faces, ranging from 0 to 360º. It determines the amount of sunlight a site receives. This may affect mosquito larval survival [12,35]. In this study, aspect was cosine-transformed so that the values ranged from −1 (south-facing slope) to +1(north-facing slope).

Curvature is a measure of the rate of change of a slope per unit distance [37]. Curvature theoretically ranges from −1 to +1 and can be categorized into profile and plan curvatures. Profile curvature is parallel to the direction of the maximum slope. A negative value indicates that the surface is upwardly convex at that cell, and a positive value indicates that the surface is upwardly concave; a value of zero indicates that the surface is linear. Profile curvature affects the acceleration or deceleration of flow across the surface. Plan curvature is perpendicular to the direction of maximum slope. A positive value indicates the surface is sidewardly convex at that cell, and a negative value indicates the surface is sidewardly concave; a value of zero indicates the surface is linear. Plan curvature relates to the convergence and divergence of flow across a surface. Considering both profile and plan curvature together allows for a more accurate understanding of flow across a surface [38,39].

Convergence index (CI) is used to determine whether water flow from neighbouring cells diverges (positive values to 100) or converges (negative values to −100). Convergence is calculated using the direction of water flow between adjacent cells based on the aspects of neighbouring cells [40].

Topographic wetness index (TWI) predicts soil moisture based on the assumption that a point with a larger upslope contribution area has greater inflow of surface water and that a point with a shallower slope has less outflow. TWI is calculated using the ratio of the upslope contributing area (A) to the tangent of local slope (tan β). Theoretically, TWI ranges from 0 to + ∞. High values of TWI are found for converging, flat terrain, while low values are typical of steep, diverging areas [41].

Topographic position index (TPI) is the deviation of a point elevation from the specified local mean, calculated by dividing the elevation difference by its standard deviation. TPI ranges from -∞ to + ∞; negative values indicate valley bottoms, while positive values signify areas such as hilltops and ridges [13,42]. Many physical and biological processes acting on the landscape are highly correlated with topographic positions such as hilltops, valley bottoms, exposed ridges, flat plains, upper or lower slopes etc. Examples of these processes include solar radiation, hydrologic balance and response, wind exposure, etc. These biophysical attributes in turn are key predictors of habitat suitability, community composition, and species distribution and abundance [42]. TPI is an inherently scale-dependent factor; thus, both a local and an area-wide scale were considered (500 m and 2000 m, respectively). The 500-m neighbourhood assists in detecting local valleys and hills, while the 2000-m neighbourhood enables identification of larger-scale features such as large U-shaped valleys, gently sloping hills, and the tops of plateaus [13].

### Statistical analyses for model development

We used logistic regression models to select variables that explained the presence and absence of anopheline larval habitats [43-45]. We first used univariate logistic regression to screen potentially important variables (*P* < 0.05), before conducting multiple regression modeling [45]. To avoid co-linearity among variables, we checked for correlation among the variables. When two variables were highly correlated with a Pearson’s correlation coefficient > 0.8, the variable with the larger Akaike information criterion (AIC) in the univariate logistic regression was removed [13]. Variables retained in the final models were selected using both forward and backward procedures (Step function in R, statistical software version 2.13 [46]). The criterion for model selection was based on the AIC [47]. All statistical analyses were performed using R 2.13. To retain a relatively simple model, second-order or higher-order interactions were not fitted. The equations generated from the logistic regression analysis for each model were applied to the topographic variables for each grid to generate risk maps for anopheline larval habitat occurrence.

We further examined the models with random variables with and without spatial autocorrelation. Description of the model and the results are shown in the Additional file 1.

### Evaluating model predictions

Assessing the predictive ability of a model is a critical step in allowing its proper applications [48,49]. We evaluated the predictiveness of our models using independent breeding site data obtained from Nyamanga in 2010. For this assessment, we employed the receiver operating characteristic (ROC) approach [50-52]. The area under the ROC curve (AUC) provides an assessment of model performance and predictive power [49]. AUC values range from 0 to 1, where a value of 0.5 indicates model accuracy not better than random and a value of 1.0 indicates a perfect model fit [50].

We also examined the applicability of our models to various types of natural breeding sites. For both the training and testing sites, the two models were separately fitted for each type of breeding site. All of the pseudo-absence points were used in each case and the AUC was used to compare the predictability of different habitat types.

### Visualization of the models

To represent the models in map form, we applied the resulting logistic regression model to the topographic variables for each grid, using the relationship:

$$p=1/\left(1+e^{-f}\right)$$

$$f=b_{0}+b_{1}x_{1}+b_{2}x_{2}+b_{3}x_{3}+\ldots +b_{n}x_{n}$$

where *b*_*0*_ is the intercept and *b*_*1*_ to *b*_*n*_ are the coefficients of the topographic variables *x*_*1*_ to *x*_*n*_, respectively. A map illustrating the *P* value was generated using the grid calculator function in the SAGA.

For easy interpretation we set cut-off values of *P* so that it divided the training site into high- and low-risk areas of nearly the same extent. Then we counted the numbers of positive sites located in the high- and low-risk sites.

## Results

### Survey results

On Rusinga Island, *Anopheles* larvae were present at 2137 aquatic sites during the survey conducted in April 2006. Of these, 1129 were in artificial containers and holes. As our purpose was to develop models to predict breeding sites from topographic variables, these artificial breeding sites were excluded in the model development. Thirty-two water bodies in tree cavities were also excluded for the same reason. GPS coordinates or habitat type information was missing for 144 sites. Ultimately, 826 natural breeding sites of 7 different types were included in the analysis (Table 1). In Nyamanga, out of the 160 potential breeding sites identified previously, many were dried up and anopheline larvae were confirmed in 54 sites of 5 types in May 2010 (Table 1).

**Table 1:** Number (%) of natural breeding sites of malaria vectors in training and testing sites

**Habitat types**	**Training site**	**Testing site**
Puddle	208 (25.1%)	13 (24.1%)
River bed	193 (23.4%)	12 (22.2%)
Drainage/ditches	169 (20.5%)	14 (25.9%)
Swamp	163 (19.7%)	9 (16.7%)
Rock pool	51 (6.2%)	6 (11.1%)
Tyre track	35 (4.2%)	0 (0%)
Foot prints	7 (0.9%)	0 (0%)
Total	826 (100%)	54 (100%)

### Simple regression analysis

The simple logistic regression analyses for both DEMs revealed that all topographic variables are significantly correlated with vector breeding sites except aspect in SRTM (Table 2). For both DEMs, breeding sites were positively correlated with TWI and negatively correlated with all other variables. For the SRTM, TWI was the best predictor of breeding sites followed by TPI_500_ and slope. For the ASTER, slope was the best predictor followed by elevation and TWI. Among all univariate models for the two DEMs, TWI in the SRTM model had the smallest AIC and thus was considered the best model.

**Table 2:** Summary of univariate logistic regression on malaria vector breeding sites with topographic variables extracted from the two different DEMs

**Variable**	**SRTM**			**ASTER**		
	**Coefficient**	**SE**	**AIC**	**Coefficient**	**SE**	**AIC**
Elevation	−0.02935 ***	0.00188	4205.0	−0.02868 ***	0.00183	4203.2
Slope	−0.2981 ***	0.01916	4176.3	−0.2595 ***	0.01589	4136.7
CosAspect	−0.11885	0.06211	4602.7	−0.28979 ***	0.06321	4584.9
Plan Curvature	−1505.78 ***	134.907	4448.0	−186.474 ***	32.1202	4572.1
Profile Curvature	−443.0664 ***	88.5638	4580.2	−41.231 (*)	21.9418	4602.9
CI	−0.052419 ***	0.00338	4327.8	−0.01649 ***	0.00305	4576.8
TWI	0.56992 ***	0.02713	4099.5	0.28397 ***	0.01459	4214.6
TPI_500_	−2.60672 ***	0.1488	4164.3	−1.78714 ***	0.10935	4255.0
TPI_2000_	−1.1472 ***	0.0748	4227.3	−1.14861 ***	0.0752	4223.7

***, P < 0.001; (*), P < 0.1.

### Correlations between predictive variables

For both SRTM and ASTER, topographic variables that were associated with occurrence of breeding sites in the simple regression were significantly correlated each other (Table 3). However, a high correlation (Pearson coefficient > 0.8) was observed only between the TPI_500_ and CI for the SRTM DEM. CI was excluded from the multivariate model with SRTM because it had larger AIC than TPI_500_ in the simple regression (Table 2). In the ASTER model, all pairs of the variables had Pearson coefficient < 0.8, and thus all 9 variables were used in the multivariate model (Table 4). TWI was negatively correlated with all of the other variables (Tables 3 and 4). The other variables were positively correlated with each other, except for slope and profile curvature, which had weak negative correlation in SRTM and ASTER (Tables 3 and 4). For ASTER, cosine aspect also showed weak negative correlations with plan curvature and profile curvature. Although these patterns were similar for SRTM and ASTER, the correlations were slightly but consistently stronger for variables from SRTM.

**Table 3:** Pearson correlation coefficients between topographic variables extracted from SRTM DEM

	**Elevation**	**Slope**	**Plan**	**Profile**	**CI**	**TWI**	**TPI**_**500**_
			**curvature**	**curvature**			
Elevation							
Slope	0.743 ***						
Plan curvature	0.399 ***	0.426 ***					
Profile curvature	0.288 ***	−0.027 **	0.332 ***				
CI	0.412 ***	0.314 ***	0.746 ***	0.437 ***			
TWI	−0.663 ***	−0.762 ***	−0.491 ***	−0.130 ***	−0.630 ***		
TPI_500_	0.538 ***	0.406 ***	0.681 ***	0.601 ***	0.875 ***	−0.664 ***	
TPI_2000_	0.681 ***	0.614 ***			0.473 ***		0.396 ***	0.590 ***	−0.714 ***	0.755 ***

Asterisks indicate level of statistical significance (** P <0.01; ***, P < 0.001).

**Table 4:** Pearson correlation coefficients between topographic variables extracted from ASTER DEM

	**Elevation**	**Slope**	**CosAspect**	**Plan**	**Profile**	**CI**	**TWI**	**TPI**_**500**_
				**curvature**	**curvature**			
Elevation								
Slope	0.650 ***							
CosAspect	0.103 ***	0.129 **						
Plan curvature	0.168 ***	0.161 ***	−0.031 *					
Profile curvature	0.198 ***	−0.017	−0.022	0.393 *				
CI	0.147 ***	0.083 ***	0.001	0.655 ***	0.388 ***			
TWI	−0.461 ***	−0.544 ***	−0.135 ***	−0.330 ***	−0.201 ***	−0.472 ***		
TPI_500_	0.548 ***	0.398 ***	0.046 ***	0.410 ***	0.426 ***	0.412 ***	−0.575 ***	
TPI_2000_	0.696 ***	0.556 ***	0.103 ***	0.214 ***	0.249 ***	0.192 ***	−0.485 ***	0.743 ***

Asterisks indicate level of statistical significance (*, P < 0.05; ** P <0.01; ***, P < 0.001).

### Multiple logistic regression analyses

Multiple logistic regressions were applied to derive models predicting potential breeding sites. The models from the two DEMs showed similar performance in terms of AIC and AUC (Tables 5 and 6). In the final SRTM model with the minimum AIC, each of the 7 variables entered were retained while in the ASTER model, CosAspect and TPI_2000_ were excluded from the final model (Table 5). The SRTM model had a slightly smaller AIC value than the ASTER model, indicating better performance of the SRTM model. The AUC of the SRTM model with the training data set was 0.758, slightly better than that of the ASTER model (0.755; Table 6).

**Table 5:** Multiple logistic regression models using SRTM and ASTER DEMs

	**SRTM (AIC = 3959.3)**		**ASTER (AIC = 3972.7)**	
	**Coefficient**	**SE**	**Coefficient**	**SE**
Intercept	−2.914 ***	0.7839	−1.913 ***	0.2379
Elevation	−0.00699 *	0.00278	−0.00841 ***	0.00223
Slope	−0.2255 ***	0.0371	−0.1215 ***	0.0204
Plane Curvature	−823.5 **	277.5	−324.9 ***	69.81
Profile Curvature	−1143 ***	270.6	90.81 *	44.88
CI	– ^a^		0.02219 ***	0.00469
TWI	0.1455 **	0.05239	0.09635 ***	0.02249
TPI_500_	−1.043 ***	0.2743	−0.9155 ***	0.1526
TPI_2000_	0.3443 **	0.1047	– ^b^	

^a^, CI was not entered to the SRTM model because of high correlation with TPI_500_.

^b^, TPI_2000_ was not selected in the final ASTER model.

Asterisks indicate levels of statistical significance (*, P < 0.05; **, P < 0.01; ***, P < 0.001).

**Table 6:** Accuracy of prediction by the two models in the training and testing sites expressed as the area under curve (AUC) of the Receiver Operating Characteristics (ROC) curve, and sensitivity and specificity

	**SRTM model**			**ASTER model**		
	**AUC**	**Sensitivity**	**Specificity**	**AUC**	**Sensitivity**	**Specificity**
		**(%)**	**(%)**		**(%)**	**(%)**
Training site	0.758	70.9	67.1	0.755	75.5	61.9
Testing site	0.829	81.5	73.9	0.799	83.3	71.8

For the testing site, both models had higher AUC scores than for the training site (Table 6). As with the training site, the SRTM model had a higher AUC value (0.829) than the ASTER model (0.799, Table 6).

When random effects was included to the models to account for spatial dependencies between close sites, the models showed better fit than those without random effect in the training sites. However, they are not better than the simple logistic models when applied to the testing site (results shown in Additional file 1).

### Applicability to different habitat types

The performance of the models in predicting the different habitat types was compared using AUC as the indicator (Table 7). For both the SRTM and ASTER models, prediction accuracy was high for drains/ditches, foot-prints puddles and swamps. On the other hand, accuracy was relatively low for rock pools and river beds. High predictability for drains/ditches and swamps was also observed at the testing site, where puddles were also predicted with high accuracy. When predictability was examined separately for each habitat type, the SRTM was not a clear improvement over ASTER.

**Table 7:** Accuracy of the model prediction of different types of breeding sites

**Site**	**Habitat type**	**N**	**AUC of Models**	
			**SRTM**	**ASTER**
Training site	Puddle	208	0.775	0.795
	River bed	193	0.632	0.698
	Drainage/ditch	169	0.796	0.824
	Swamp	163	0.915	0.895
	Rock pool	51	0.559	0.681
	Tyre track	35	0.708	0.809
	Foot print	7	0.911	0.975
Testing site	Puddle	13	0.939	0.983
	River bed	12	0.644	0.731
	Drainage & ditch	14	0.839	0.927
	Swamp	9	0.948	0.969
	Rock pool	6	0.761	0.737

### Visualization of the models

For both the SRTM and ASTER models, the visualized maps showed good fitting with the observed locations of breeding sites.

**Figure 2:**
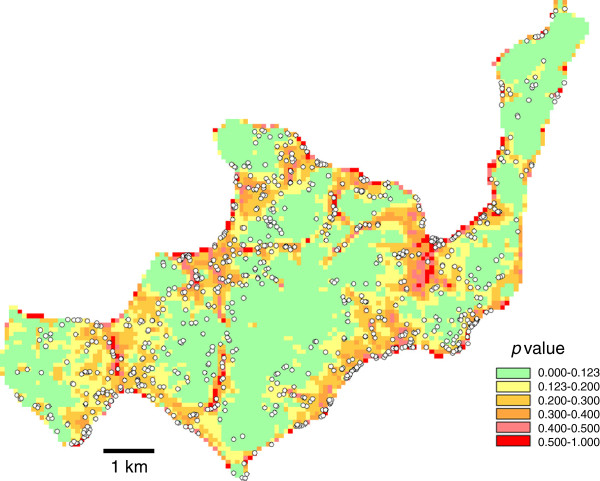
**SRTM model: the likelihood of the presence of breeding sites in Rusinga based on logistic regression modeling with the topographic variables presented in Table**5**.** Observed breeding sites are indicated with white dots.

**Figure 3:**
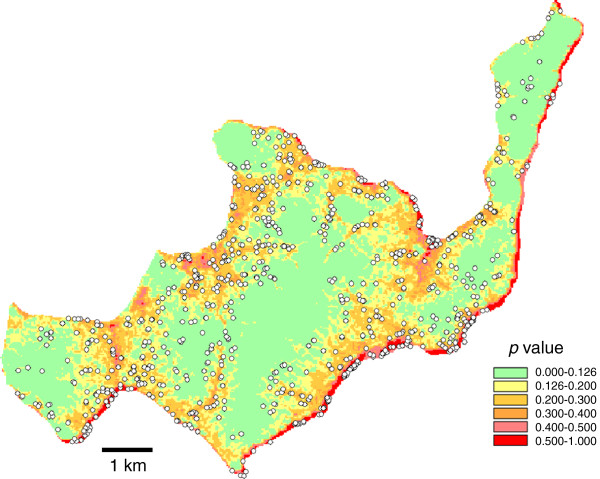
**ASTER model: the likelihood of the presence of breeding sites in Rusinga based on logistic regression modeling with the topographic variables presented in Table**5**.** Observed breeding sites are indicated with white dots.

For the SRTM model, the high risk area with *p* > 0.123 made up 49.8% of the total area of the island, and contained 658 (79.7%) breeding sites (Figure 2). The high-risk areas with *p* > 0.130 in the ASTER model made up 49.7% of the total area and contained 675 (81.7%) breeding sites (Figure 3).

**Figure 4:**
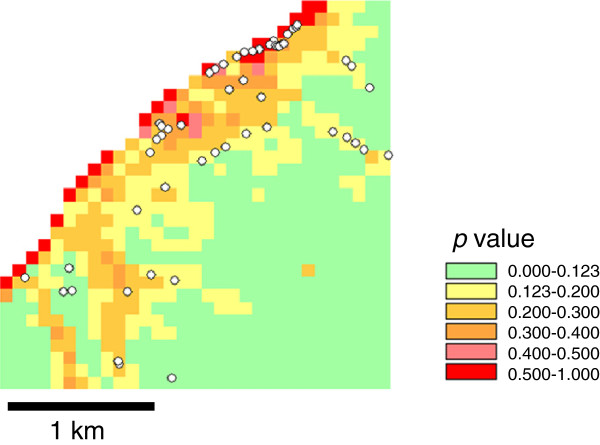
**SRTM model: the likelihood of the presence of breeding sites in Nyamanga based on logistic regression modeling with the topographic variables presented in Table**5**.** Observed breeding sites are indicated with white dots.

**Figure 5:**
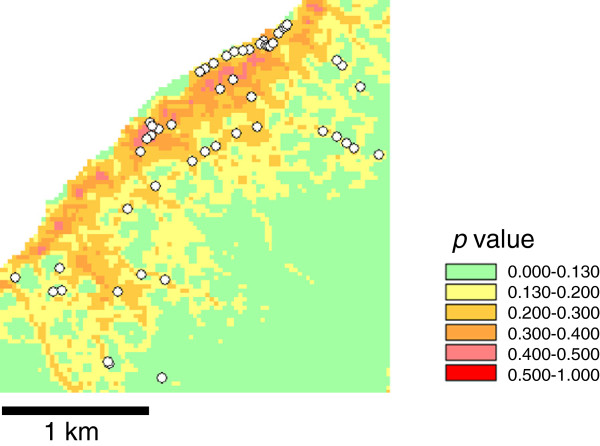
**ASTER model: the likelihood of the presence of breeding sites in Nyamanga based on logistic regression modeling with the topographic variables presented in Table**5**.** Observed breeding sites are indicated with white dots.

High accuracy of the model prediction in the testing site was indicated visually in the maps (Figures 4 and 5). The area with *p* > 0.123 in the SRTM model made up 44.7% of the total area and contained 47 (88.0%) breeding sites (Figure 4). For the ASTER model, the high-risk areas with *p* > 0.130 made up 48.1% of the total area and contained 48 (88.9%) breeding sites (Figure 5).

## Discussion

In this study, we developed practical models for predicting malaria vector breeding sites using topographic variables. The two models using different DEMs had similar performance and their accuracies were quite good for both training and testing sites. The present study confirmed that the use of multiple topographic variables in combination is effective for predicting larval habitats of malaria vectors. Elevation and slope have direct effects on surface water flow, since water flows from high to low elevations. More complex variables such as plan and profile curvatures, CI, TWI, and TPI are also related to surface water flow. These variables are correlated, but somewhat different from each other. Thus the multivariate logistic model performed much better than did the univariate models. Previous studies using both topographic and land-cover variables have succeeded in predicting vector breeding sites with high accuracies. For example, one of the models developed by Clennon *et al.*[13] had an AUC >0.95. The accuracy of our models was not as high as this. However, our model was able to indentify high-risk areas that made up about half of the total area but included nearly 80% of the breeding sites, which would be helpful to reduce target areas for vector control. We consider this an acceptable level of accuracy for predicting breeding sites.

Our model has fundamental practical advantages over the previous studies. First, because our models require only freely available DEMs that cover most of the land surface area (all of tropical area), they can be applied even in places where satellite images of good quality are not available. Furthermore, the present analyses were mainly conducted using free software. This permits easy evaluation and application of our models to a prospective study area. The only necessary resources are a personal computer, an internet connection, and moderate skill in use of the computer. This is an economic advantage of our models, particularly in countries with limited resources. Second, our models were developed using the results of an extensive survey over the entire area of a large island (42 km^2^). The extent of our study site was larger than that of previous studies [12,13]. Given that the island consists of a range of topographic features and the survey identified various types of breeding sites, we expect that our models may be applicable to a variety of environments with differing topography and with different types of natural breeding sites. Third, in the present study we tested the accuracy of the model predictions in an independent area and confirmed good performance. Assessing the predictiveness of models in an independent area from the training site is considered the best approach for model testing and for evaluating model applicability [48,49]. Previous studies did not examined model performance using independent data sets [13,16].

It is of interest how far our model can be applied to the area of different geographical settings. The absolute probability of water pooling in an area of a certain topographic feature must be different in areas with different levels of precipitation and different soil types. However, it is possible that the topographic models can predict relative likelihood of water retention as far as water is more likely to retain in valley bottoms or plains near the foot of a mountain than in steep slopes. We hope that our model will be tested in different geographical settings for further validation.

It would be expected that breeding sites could be predicted more accurately with higher-resolution data, holding the other conditions constant. However, in the present study, the lower-resolution (90-m) SRTM DEM performed slightly better than the higher-resolution (30-m) ASTER DEM. One possible reason for this is a difference in the method used to measure elevation: SRTM measures elevation by receiving a radar signal that bounces off the earth’s surface while, ASTER estimates elevation by comparing two optical images taken at a certain interval. SRTM is the more direct measurement and thus should be more accurate if the resolution is the same. The elevation of the surface of Lake Victoria was identified as 1134 m in SRTM and varied from 1126 to1128 m in ASTER. The former appears to be closer to the actual elevation of Lake Victoria [53]. This result agrees with Clennon *et al.*[13], who reported that information derived from SRTM performed better than ASTER data in predicting breeding sites.

It was also unexpected that the accuracy of our models was better at the testing site with independent data than at the training site where the models were developed. This appears to be a deviation from the norm, because models are optimized to the training site. One possible explanation is that a large percentage of the southern test area was sloped and mountainous, and thus was predicted as a low risk-area. Breeding sites were concentrated near the lake shore, and model performance may be higher in such geographic settings. Another possible reason could be different climate conditions in the two study periods. Survey on the training site was carried out in the peak of the rainy season (April) of 2006; whereas the testing site was surveyed towards the end of the rainy season (May) of 2010. Considerable portions of the potential breeding sites were dried up in May 2010, as there was little rainfall during 10 days prior to this survey (unpublished data). It is possible that only stable habitats remained in the testing sites in the survey period and they are relatively easy to predict.

The predictiveness of our models varied with habitat type. Although the predictability was better than random for all habitat types, drainage/ditches, puddles, footprints, swamps, and tire tracks were highly predictable while rock pools and river beds were less predictable. High predictability for tire tracks and footprints could be related to the models’ ability to predict flat areas in low-lands where small depressions in the land surface such as foot-prints and tire tracks are likely to retain water. These sites may have similar topographic features to swamps, and are likely to occur around the fringes of swamps. These results suggest that our models are most useful if the main breeding sites are swamps and foot-prints. Low predictability of riverbed was unexpected, because riverbed pools are formed along drying streams, which would be easily predicted by topography. When we displayed the riverbed habitats on the maps, they are not well located along the river channel. We suspect that miss-classification of this type of habitat is the reason of low predictability of riverbed.

There are some limitations to the present study. First, because we did not use land-cover information for simplicity, predictive power may have been limited. This would be particularly important in areas where the vector species prefers specific land-cover types and the land-cover types in the area of interest are heterogeneous. Both the training and testing sites in the present study had been entirely deforested and thus had relatively homogeneous landscapes. In such areas, land-cover would not be an important limiting factor for the vectors. This may be one possible reason for the high accuracy of our models. Similarly, if the target area consists of sub-areas of different soil types that differ in capacity of holding water, predictability of our models would be limited. When these problems with land-cover and soil types seriously limit the predictability of simple topographic models, the use of satellite imagery should be considered as in the previous studies [12,13].

Second, we used the results of an extensive survey that was carried out once in the rainy season (April). Therefore, our model may predict breeding sites well in the rainy season, but not in the dry season. Given that the locations of breeding sites would likely be different in the rainy and dry seasons [12,54], another model may be necessary to predict dry season breeding sites.

Third, because we prioritized model simplicity, we assumed linear relationships between predictors and the logit of likely presence and did not consider any non-linear relationships or interactions among variables. For example, because mosquito larvae never occur in either dry soil or fast running water, a unimodal relationship may occur between larval habitat and certain indices such as TWI over a wide range. It is possible that the simplicity of our model sacrificed predictability to some extent.

Fourth, our survey did not distinguish species of *Anopheles* mosquitoes. Because different species may prefer aquatic sites of different topographic features, the fit of the model would be greatest when each species was treated separately. In our models, the relationship between topographic features and the presence of larval habitats would be less clear compared with the species specific models. In the study area, *Anopheles gambiae* species complex (*A*. *gambiae* and *A*. *arabiensis*) were found as the majority of the larval samples and *A*. *funestus* occurred less frequently in the rainy season. Application of our models to areas with different vector species should be conducted with caution.

Fifth, since we did not record aquatic sites without *Anopheles* larvae we could not model formation of aquatic sites. Formation of standing water on the surface ground is purely a physical process, so should be best predicted by topographic variables. Occurrence of *Anopheles* larvae may also depend on biological factors, such as water quality, occurrence of predators, and proximity to the blood meal, etc. When the observed habitats were overlaid with the model projections, (Figures 2, 3, 4 and 5), some areas are shown with high risk but no habitats. These might be aquatic sites that are not inhabited by anopheline larvae. It is desirable to have two different models that predict aquatic sites and anopheline habitats.

Lastly, it should be noted that the topographical model has a fundamental limitation when man-made habitats are important breeding sites of the vectors. Our survey on Rusinga found that more than half of the total numbers of breeding sites were artificial ones, such as holes and containers. These breeding sites might be important especially in urban environments and an alternative way to predict breeding sites would be necessary in such a situation.

Despite these limitations, our study adds confidence to localized mosquito habitat management and provides simple solutions for habitat modeling. We have demonstrated the feasibility of predicting potential breeding habitats of *Anopheles* mosquitoes using topographic variables derived from freely available DEMs. Our models are expected to be useful in defining target areas for larval control. Furthermore, application of these models to large areas may help identify high-risk areas for malaria infection, because it is most likely that malaria prevalence would be higher in areas with many potential breeding sites of malaria vectors [55,56].

## Conclusion

This study has demonstrated that with the advent of freely available SRTM and ASTER DEMs, topographic models that predict malaria vector breeding habitats could be developed. These models could be more practically and widely used to complement targeted malaria control strategies. In particular, these maps can help exclude areas where breeding sites are unlikely to be present, and so help prioritize high risk areas more precisely. Targeted larval control would greatly maximize limited resources and thus, should be strongly considered in integrated malaria management in Africa.

## Competing interests

The authors declare that they have no competing interests.

## Authors’ contributions

JCN was the principal investigator and was responsible for the study design, data analysis, interpretation, and writing of the manuscript. TS, KG, KF, and NM assisted in the study design, data analysis and interpretation. PA, GS, GD, KF, and NM assisted with the field-work. TS, KG, and NM revised the manuscript for intellectual content. TS, KG, and NM supervised the work. All authors read and approved the final manuscript.

## Supplementary Material

Additional file 1 Models with random effects with and without spatial autocorrelation. Click here for file
