# Supplementary material for: Topographic models for predicting malaria vector breeding habitats: potential tools for vector control managers
Source: Parasit Vectors. 2013 Jan 16;6:14. doi: 10.1186/1756-3305-6-14 (PMC3617103; doi:10.1186/1756-3305-6-14)
Supplement: Additional file 1 — Models with random effects with and without spatial autocorrelation. [file 1756-3305-6-14-S1.pdf]

Additional file 1

Models with random effects with and without spatial autocorrelation

When spatially structured factors not considered in the model influence the formation of the breeding sites, the fit of the models could be improved by adding random variables with spatial autocorrelation. Although random variables cannot be used in the model prediction for areas outside the training site, the effects of topographic variables may be estimated more precisely by adjusting the random variables. Therefore, we compared the predictive power of three kinds of models: fixed model, mixed model with a random factor, and mixed model with a spatially autocorrelated random factor. Because models with a spatially autocorrelated random variable can be treated only by Bayesian statistics, the analysis was carried out in the Bayesian framework by using WinBUGS, version 1.4.3 [A1] with R2WinBUGS package of R 2.15.

We first examined whether the residuals of the logistic models in Table 5 were spatially auto-correlated by using Moran's I as the indicator. Neighbor points were defined in different scales from 100 m to 1000 m by using the *dnearneigh* function in the *spdep* package of R with row standardized weights (style = "W" in nb2listw function). A randomization test performed using *moran.test* indicated highly significant positive autocorrelation over the entire spatial scale examined in both the SRTM and ASTER models.

To define random variables, we constructed a 250 m × 250 m grid system that covers the whole of Rusinga Island. This resolution was chosen so that most of the grids contain multiple points (either positive or pseudo-negative). Out of the 744 grids, 2 (0.26%) contained no points, 32 (4.4%) contained single points, and 710 (95.4%)

contained multiple (2–19) points. The mean and the standard deviation of the number of points per grid were 7.18 and 3.19, respectively. We defined two kinds of random variables: first, random variables for each grid independent from each other; second, random variables for each grid correlated with the values in the neighbor grids.

The models with same topographic variables selected in the logistic models with SRTM and ASTER DEMs (Table 5) were examined. The fix models were equivalent to the models in Table 5. In the mixed model, we added a random variable r_n for each grid that follows normal distribution with the mean zero and variance s_n. In the spatial autocorrelation model, we set random variable r_s so that the values of adjacent grids show a certain level of correlation, using the car.normal() function of WinBUGS [A2]. The value r_s follows normal distribution with mean zero and variance among adjacent cells, s_s.

Prior distribution for the coefficients for intercepts and topographic variables were set as non-informative uniform distribution by using the dflat() function, except for the coefficient of TWI. Because the coefficients for TWI were barely converted with dflat() prior distribution, we arbitrarily set the prior distribution of TWI coefficients as normal distributions in which the mean was equal to the coefficients estimated in logistic model (Table 5; 0.145 for SRTM and 0.096 for ASTER) and variance is 0.2. The criteria for convergence of the models were as follows: (1) R-hat statistic (Brooks-Gelman-Rubin statistic in WINBUGS) is less than 1.1, and (2) the effective sample size of the chains (n.eff statistic in R) is greater than 100 for all parameters [A3].

In both models with SRTM and ASTER, the addition of the random variable greatly improved the fit of the model, as shown by the smaller DIC in the mixed model than in the fixed model (Table A1 and A2). When the spatial autocorrelation of the

random variables was considered, the fit of the model was further improved. However, the coefficient values for each variable did not differ greatly among the three models. When only using the coefficients of the intercept and fixed variables, the prediction accuracies of the mixed model and spatial autocorrelation model were not better (SRTM) or only slightly better (ASTER for the test site) than the that of the fixed models (Table A1 and A2). Thus, we conclude that the simple logistic model is sufficient for our purpose.

References

A1. D J Spiegelhalter DJ, Thomas A, Best NG. WinBUGS Version 1.4 User Manual. Imperial College and Medical Research Council, UK. 2003 http://www.mrc-cam.ac.uk/bugs

A2. Thomas A, Best N, Lunn D, Arnold R, Spiegelhalter D. GeoBUGS User manual Version 1.2. Medical Research Council Biostatistics Unit, University of Cambridge, UK. 2004. http://www.mrc-bsu.cam.ac.uk/bugs.

A3. Brooks SP, Gelman A. **General methods for monitoring convergence of iterative simulations.** J. Comp. Graph. Statist. 1998; 7: 434-55

Table A1. Comparison of the coefficients and predictability of the three different models with SRTM DEM.

	Fixed model		Mixed model		Spatial model	
	Mean	95% range	Mean	95% range	Mean	95% range
Coefficients						
Intercept	-2.794	(-4.277, -1.172)	-4.761	(-6.967, -2.634)	-4.307	(-7.142, -1.682)
Relative elevation	-0.00702	(-0.0125, -0.0016)	-0.00946	(-0.0179, -0.0012)	-0.01313	(-0.031, 0.004)
Slope	-0.230	(-0.305, -0.159)	-0.271	(-0.374, -0.176)	-0.315	(-0.424, -0.209)
PlanCurv	-828.9	(-1372.0, -287.9)	-1147.9	(-1882.0, -429.6)	-1095.4	(-1834.02, -370.1)
ProfCurv	-1148.6	(-1685.0, -622.7)	-1500.5	(-2197.0, -824.3)	-1409.4	(-2104.0, -731.4)
TWI	0.138	(0.030, 0.237)	0.269	(0.126, 0.416)	0.260	(0.085, 0.446)
TPI500	-1.059	(-1.006, -0.531)	-0.874	(-1.655, -0.097)	-0.837	(-1.675, 0.000)
TPI2000	0.341	(0.132, 0.546)	0.509	(0.196, 0.817)	0.712	(0.142, 1.274)
Random variables						
s_n	--		0.589	(0.454, 0.779)	--	
s_s	--		--		7.992	(5.917, 10.753)
DIC	3959.2		3551.6		3526.6	
AUC in the training site*	0.759		0.757		0.754	
AUC in the test site*	0.827		0.826		0.824	

*Random effects were not included in the assessment of predictive power.

Table A2. Comparison of the coefficients and predictability of the three different models with ASTER DEM.

	Fixed model		Mixed model		Spatial model	
	Mean	95% range	Mean	95% range	Mean	95% range
Coefficients						
Intercept	-1.909	(-2.369, -1.446)	-2.569	(-3.274, -1.888)	-2.590	(-3.395, -1.8)
Relative elevation	-0.00844	(-0.013, -0.004)	-0.01367	(-0.02, -0.007)	-0.01727	(-0.028, -0.007)
Slope	-0.123	(-0.164, -0.084)	-0.123	(-0.173, -0.074)	-0.125	(-0.178, -0.073)
Plane Curvature	-324.7	(-460.6, -187.948)	-396.6	(-554.7, -238.5)	-394.8	(-551.50, -237.60)
Profile Curvature	92.8	(6.451, 182)	90.6	(-15.053, 196.9)	84.3	(-21.401, 193.6)
Convergence Index	0.022	(0.013, 0.031)	0.028	(0.016, 0.039)	0.027	(0.016, 0.039)
TWI	0.096	(0.052, 0.14)	0.143	(0.079, 0.209)	0.162	(0.09, 0.236)
TPI500	-0.920	(-1.225, -0.619)	-0.829	(-1.249, -0.412)	-0.546	(-1.005, -0.088)
Random variables						
s_n	--		0.773	(0.676, 0.890)	--	
s_s	--		--		8.052	(6.017, 10.941)
DIC	3972.7		3577.6		3526.2	
AUC in the training site*	0.755		0.753		0.750	
AUC in the test site*	0.820		0.826		0.830	

*Random effects were not included in the assessment of predictive power.
